# Comparing determinants of physical activity in Puerto Rican, Mexican-American, and non-Hispanic white breast cancer survivors

**DOI:** 10.1186/s40064-015-1190-5

**Published:** 2015-08-13

**Authors:** Daniel C Hughes, Maribel Tirado-Gomez, Liliana Vallejo, Velda Gonzalez, Rose A Treviño-Whitaker, Gabriela Villanueva, Karen Basen-Engquist

**Affiliations:** Institute for Health Promotion Research, The University of Texas Health Science Center at San Antonio, 7411 John Smith Drive, Suite 1000, San Antonio, TX 78229 USA; The University of Puerto Rico Cancer Center, San Juan, Puerto Rico; Department of Behavioral Science, The University of Texas M.D. Anderson Cancer Center, Houston, USA

**Keywords:** Hispanics, Social cognitive theory, Exercise, Breast cancer

## Abstract

**Purpose:**

Physical activity (PA) has a myriad of benefits for breast cancer survivors, including a reduced risk of cancer recurrence. Latinas are less physically active than are women in the general population and little is known about Latina breast cancer survivors’ levels of PA or their beliefs related to PA. We conducted a survey of 50 Puerto Rican (PR), 50 Mexican-American (MA) and 50 non-Hispanic white (NHW) breast cancer survivors to investigate similarities and differences in PA and social cognitive theory (SCT) constructs associated with PA.

**Methods:**

We collected information on current PA using the Godin Leisure Time Exercise Questionnaire (GLTEQ); comorbidities; anthropometric measures of body mass index [BMI (kg/m^2^)] and waist-to-hip (W:H) ratio; and SCT measures, including exercise self-efficacy, exercise barriers self-efficacy, modeling and social support from friends and family. Descriptive statistics, one-way analysis of variance of differences between groups and regression models of the predictors of PA were performed.

**Results:**

Survivors from the three groups were similar in age (M = 56.8, SD = 11.0), BMI (M = 29.0, SD = 5.7) and co-morbidity (M = 2.09, SD = 1.69). Survivors differed in PA (*p* < 0.001), self-efficacy (*p* = 0.05), modeling (*p* = 0.03) and social support from family (*p* = 0.05). Social support from family member and exercise barriers self-efficacy were predictors of PA.

**Conclusions:**

Consistent with published studies, Hispanic breast cancer survivors self-report that they are less physically active than are non-Hispanic whites. SCT variables associated with PA differ among Hispanic subgroups and non-Hispanic whites. Further research is warranted in order to understand determinants of physical activity for specific ethnic breast cancer survivors.

## Background

The US now has more than 12 million cancer survivors (American Cancer Society 2012) and at least 1.8 million of those are Hispanic (American Cancer Society 2009). Hispanics are the most rapidly growing ethnicity in the United States (United States Census Bureau 2000), and breast cancer is the leading cause of cancer death among Hispanic women (American Cancer Society 2009). In Puerto Rico it accounts for approximately 34% of all female cancers and is the most common female malignancy diagnosed (Puerto Rico Central Cancer Registry 2007).

Research continues to document the benefits of exercise for breast cancer survivors, including improved fitness, physical functioning, fatigue and emotional well-being (Courneya 2003; Courneya et al. 2003; Segal et al. 2001; Pinto et al. 2005). Moreover, cohort studies have shown a decreased risk of breast cancer recurrence and reduced mortality among survivors who are more active (Holmes et al. 2005; Irwin et al. 2011; Ballard-Barbash et al. 2012; Patterson et al. 2010). However, only a small percentage of survivors are active at levels consistent with public health guidelines (Schmitz et al. 2010). Like others who experience cancer, many breast cancer survivors who were not active before diagnosis will remain inactive, and those who were active often do not return to their previous level of activity (Schmitz et al. 2010). In fact, approximately four of every five breast cancer survivors do not meet national exercise recommendations at 10 years post-diagnosis (Mason et al. 2013). Little is known about the physical activity levels of Hispanic breast cancer survivors, although some studies indicate that in the general US population, Latinas report higher rates of inactivity than do non-Hispanic white women (Castro et al. 1999; Crespo et al. 2000).

Although the benefits of physical activity for cancer survivors continue to be documented and are becoming better understood, research is needed to identify interventions that encourage cancer survivors to begin and/or maintain consistent exercise patterns. Intervention strategies for healthy and predominately non-Hispanic white populations may not apply to members of specific ethno-cultural populations that have experienced cancer and who may face additional cultural factors that affect physical activity. Furthermore, diversity within Hispanic populations may also affect the likelihood that an individual will pursue physical activity after cancer diagnosis and treatment. Differences among Hispanic groups, including race, country of origin, levels of acculturation, English language proficiency, and other cultural variables, may affect health-promoting behaviors, including exercise.

Therefore we believe that investigating the key variables that affect physical activity adoption and maintenance within specific cultural contexts of Hispanic breast cancer survivor groups is a high priority for behavioral research. Understanding the knowledge, attitudes and barriers that surround physical activity among Hispanic groups is fundamental in the development of culturally appropriate exercise interventions. To provide formative data for adapting a culturally sensitive social cognitive theory (SCT)-based exercise intervention, we conducted a cross-sectional survey to investigate similarities and differences in SCT, co-morbidity and anthropometric variables associated with current level of physical activity in Puerto Rican, Mexican-American and non-Hispanic white breast cancer survivors.

## Methods

A sample of 150 breast cancer survivors completed an interviewer-guided survey. Fifty Puerto-Rican (PR) participants completed the survey in San Juan Puerto Rico at the Oncologic Hospital Dr. Isaac Gonzalez Martinez. Fifty Mexican-American (MA) and 50 non-Hispanic white (NHW) breast cancer survivors completed the survey at The University of Texas M.D. Anderson Cancer Center (UTMDACC). The research protocol was approved by the institutional review board of UTMDACC. In Puerto Rico, the research protocol was approved by the University of Puerto Rico Medical Sciences and the institutional review board of the Oncologic Hospital Dr. Isaac Gonzalez Martinez. Participants were invited to participate in the study if they: (1) had a diagnosis of invasive breast cancer or ductal carcinoma in situ; (2) were older than 21 years (Puerto Rico) or older than 18 years (UTMDACC) of age; (3) had finished their chemotherapy and/or radiotherapy at least four months before the date of the survey (hormonal therapy was allowed); and, (4) had no evidence of metastatic disease.

For Puerto Rican participants, the clinical study coordinator reviewed the medical charts of patients attending the outpatient clinic in the Oncologic Hospital Dr. Isaac Gonzalez Martinez clinic and then met with potential participants who met the inclusion criteria at the clinic. Participants who expressed interest in participating received an informative sheet describing the study’s purpose and procedures. Verbal consent was obtained before data were collected. Participants filled out the questionnaires in the presence of the clinical study coordinator who was available to answer any questions. Surveys were conducted in Spanish.

At UTMDACC, women were recruited through the Breast Oncology Clinic during scheduled appointments or from Houston community breast cancer support groups. From UTMDACC patient data records, a list of women who met the inclusion criteria and were returning for follow-up care was generated. These women received a letter in the mail that provided information about the study and with a number to call if not interested. If no call was received, a bilingual research coordinator called and/or met the patient at her follow-up appointment to explain the study, obtain verbal informed consent and schedule an interview. Participants at UTMDACC were asked whether they preferred Spanish or English. The survey questions were interviewer guided. To help facilitate the interviewer-guided response, participants were given response cards specific to each section of the questionnaire, (e.g., Likert scale choices in large print). The research coordinator explained the use of response cards. Participants were asked to give their initial response after each item was read aloud. For both UPRCC and MDACC, the survey included identical questions on demographic/personal information, health history, level of physical activity and SCT variables.

Demographic and personal information included living location; self-reported ethnicity/race; education; marital status; self-reported height and weight (used for BMI calculation) and medical history. A co-morbidity index was calculated using responses from the medical history information. A total of 17 items were included: diagnosis of a heart attack, heart failure, heart condition, circulation problems, blood clots, hypertension, stroke, lung problems, diabetes, kidney problems, rheumatoid arthritis, osteoarthritis, anemia, thyroid problems, neuropathy, fibromyalgia and hepatitis. The co-morbidity index was calculated by adding the number of individual responses marked as “Yes” (with a possible range of scores of 0–17). Participants also were whether they experienced any secondary cancers and whether they had been given a diagnosis of lymphedema (The information on secondary cancers and lymphedema was noted but was treated separately from the co-morbidity index).

Current level of physical activity (PA) was assessed with The Godin Leisure Time Exercise Questionnaire (GLTEQ) (Godin and Shephard 1985). The GLTEQ measures leisure time exercise behaviors for a typical week. Participants completed a four-item questionnaire of usual leisure-time exercise habits. As an example, a typical question regarding strenuous activity was: “Considering a 7-day period (a week), how many times, on the average, do you do the following kinds of exercise for more than 15 min during your free time: a) strenuous exercise (hearts beats rapidly [i.e., running, jogging, football, soccer, squash, basketball, cross country skiing, judo, roller skating, vigorous long distance bicycling.])”. The weekly frequency of each participant’s strenuous, moderate and light activities were multiplied by 9, 5 and 3, respectively which is an approximate metabolic equivalents (MET) value for that level of activity. MET is the metabolic equivalent of the level of energy consumption for a body at rest; the higher a MET value, the higher the energy requirement. The MET value was used with the individual’s self-reported frequency to calculate a weekly activity score [weekly activity score = (9 × “strenuous”) + (5 × “moderate”) + (3 × “light”)]. The GLTEQ has been validated as a means of discriminating among levels of PA and has compared well with other measures of exercise, fitness indices, and physical activity monitors (Godin and Shephard 1985).

SCT variables of self-efficacy, modeling and social support were included in the survey. Self-efficacy was measured with a questionnaire based on one used by McAuley (1993); (McAuley et al. 1994, 2003; Duncan and McAuley 1993) that assesses confidence in sustaining various durations of a specific exercise, in our case, walking. A typical question was: “How confident are you that you can…Walk briskly without stopping for 5 min?” The responses range from 1 = “not at all confident” to 5 = “extremely confident”. The range of the time frame we used for minutes of walking was from 2 min to 1 h (2, 5, 10, 20, 30, 45 min, 1 h). Responses to the seven individual items were summed to obtain an overall score with a possible range of 7–35. Internal consistency (Cronbach’s alpha) for our survey was *r* = 0.96.

To measure exercise barriers self-efficacy we adapted a questionnaire developed by Marcus et al. (1992). After pilot testing the questionnaire, we added items specific for cancer survivors and arrived at a 14 point scale (Basen-Engquist et al. 2009, 2013). A typical question was: “How confident are you that you can exercise…When you are concerned about your medical condition?” Participants responded to a level of confidence ranging from 1 = “not at all confident” to 5 = “extremely confident”. Responses to the 14 individual items were summed to obtain an overall score with a possible range of 14–70. Internal consistency (Cronbach’s alpha) for our survey was *r* = 0.83.

We assessed social support for exercise by adapting Sallis’ Social Support and Exercise Survey (Sallis et al. 1987) to a 10-item survey. Participants were asked about the degree of support for exercise that they receive from family/friends. A typical question was: “During the past 3 months, my family (or members of my household) helped plan activities around my exercise”. Response categories included: 1 = “none”, 2 = “rarely”, 3 = “few times”, 4 = “often” and 5 = “very often”. We analyzed responses related to social support from family separate from social support from friends. Responses to the individual items were summed to obtain an overall score with a possible range of 10–50. Internal consistency (Cronbach’s alpha) for our surveys were *r* = 0.90 and *r* = 0.94 for social support from family and friends, respectively.

Modeling of physical activity was assessed with eight questions that asked the participants to indicate whether they observed people in their social environment or the media engaging in or discussing physical activity/exercise. Participants were asked to respond “yes” or “no” to the following questions: “Today, the following happened: (1) I noticed people like me exercising; (2) A friend or family member offered to exercise with me; (3) I read or heard news stories about people exercising; (4) I was aware that a member of my family exercised today; (5) A friend or family member exercised with me; (6) A friend or family member talked to me about their exercise program; (7) A friend or family member shared their experience about how to stay with an exercise program; and (8) I noticed people walking in my neighborhood.” The total number of “yes” responses was added to obtain a total score with a possible range from 0 to 8. Internal consistency (Cronbach’s alpha) for our survey was *r* = 0.75.

Upon completion of the questionnaires, height (m), weight (kg), waist circumference (cm) and hip circumference (cm) were measured for each participant. Height (m) and weight (kg) data were used to calculate BMI (kg/m^2^). (At UPRCC, self-reported height and weight were used for BMI calculations instead of being measured). Waistline measurements were taken at the midpoint between the lowest rib and the iliac crest, at the iliac crest, at the narrowest point of the waist area, over the umbilicus area, and at the widest part of the hips. When the tape was accurately placed on the anatomical site against the skin, the participant was asked to inhale and exhale. The measurement was then taken in inches after the participant exhaled. Inches were converted to cm for the narrowest part of the waist and for the widest part of the hips. A waist-to-hip (W:H) ratio was calculated.

### Treatment of data

All data were entered and analyzed using SPSS V 20.0 (Statistical Package for the Social Sciences), (SPSS, Inc., Chicago, Ill, USA). Descriptive statistics were calculated for participant demographics, activity levels, SCT variables, and anthropometric measures. To compare mean differences among the three groups, we conducted a one-way analysis of variance (ANOVA) with a post-hoc Tukey analysis. We then ran a two-step regression model for current level of activity (GLTEQ), first with demographic variables (education, age, and ethnic group) and then with the SCT variables (exercise barriers self-efficacy, exercise self-efficacy, social support from family, social support from friends, and modeling) added as predictors; changes in R^2^ were calculated. We then ran models that added the interaction terms between each SCT variable and ethnic group. Statistical significance for ANOVA differences and regression analyses was set at *p* > 0.05.

## Results

Participant demographic and health characteristics are shown in Table 1. There were no differences among groups in age, BMI and co-morbidity index. Averaged together our participants averaged 56.8 years of age, an overweight BMI of 29.0 kg/m^2^ and moderate level of 2.1 co-morbidities when measured with our index. The NHW group was more educated than either the PR or MA subgroups, with more NHW participants reporting being employed. Waist-to-hip ratio were similar for PR and MA (M = 0.83, M = 0.82, respectively) and lower for NHW (M = 0.79).

**Table 1:** Comparison of demographic and health variables for Puerto Rican, Mexican-American and non-Hispanic white breast cancer survivors

	Overall, M (SD)	PR, M (SD)	MA, M (SD)	NHW, M (SD)	*p*
Age	56.8 (11.0)	57.2 (12.2)	54.7 (9.3)	58.5 (11.1)	0.193
BMI	29.0 (5.7)	29.4 (3.8)	29.8 (7.0)	27.7 (5.5)	0.138
Waist-to-hip ratio	0.81 (0.06)	0.83 (0.06)	0.82 (0.06)	0.79 (0.05)	0.003*
Comorbidity	2.1 (1.7)	2.3 (1.6)	1.9 (1.4)	2.1 (2.0)	0.450

	N (%)	N (%)	N (%)	N (%)
Education				
No high school	31 (20.7)	14 (28.0)	17 (34.0)	0 (0.0)
High school	23 (15.3)	12 (24.0)	6 (26.1)	5 (10.0)
Some college	44 (29.3)	8 (16.0)	16 (32.0)	20 (40.0)
Bachelor’s degree	28 (18.7)	11 (22.0)	8 (16.0)	9 (18.0)
Post bachelor’s degree	24 (16.0)	5 (10.0)	3 (6.0)	16 (32.0)
Employment status				
Full time	58 (38.9)	13 (26.0)	21 (42.0)	24 (48.0)
Part time	10 (6.7)	0 (0)	7 (14.0)	7 (6.0)
Not employed for pay	14 (9.4)	6 (12.0)	6 (12.0)	2 (4.0)
Homemaker	39 (26.2)	20 (40.0)	13 (26.0)	6 (12.0)
Retired/volunteer	28 (18.8)	10 (20.0)	3 (6.0)	13 (26.0)
No answer	1	1		

*PR* Hispanic Puerto Rican participants, *MA* Hispanic Mexican-American participants, *NHW* non-Hispanic white participants, *SD* standard deviation.

* PR > NHW, (p = 0.006); MA > NHW, (p = 0.026).

Results of analyses of frequencies of intensity levels of PA and calculated GLTEQ scores are shown in Table 2. Differences are apparent in the GLTEQ score (*p* < 0.001) with the PR group having the lowest levels (M = 12.9, SD = 17.6), MA higher than PR level (M = 31.4, SD = 18.7) and NHW demonstrating the highest levels (M = 40.9, SD = 25.9) of the three groups. The differences remained significant after controlling for age, education, and employment status (p < 0.001). Seventy-six percent of PR survivors reported doing no moderate or strenuous activity compared to 22% and 18% of MA and NHW participants, respectively.

**Table 2:** Descriptive statistics for Godin Leisure Time Activity Scale by ethnic group

	Puerto Rican	Mexican-American	Non-Hispanic white
Light activity frequency			
% with no activity	62	18	18
Quartiles	0, 0, 3	1, 4, 7	1.75, 3, 7
Mean (SD)	1.76 (2.49)	3.82 (2.70)	3.60 (2.63)
Moderate activity frequency			
% with no activity	76	28	26
Quartiles	0, 0, 0.25	0, 2, 3	0, 2.50, 5
Mean (SD)	1.06 (2.20)	2.12 (1.85)	2.78 (2.30)
Strenuous activity frequency			
% with no activity	94	60	44
Quartiles	0, 0, 0	0, 0, 2	0, 1, 3
Mean (SD)	0.26 (1.06)	1.04 (1.67)	1.8 (1.95)
Moderate + strenuous activity frequency			
% with no activity	76	22	18
Quartiles	0, 0, 0.25	1, 3, 5	2, 4, 7
Mean (SD)	1.32 (2.64)	3.16 (2.63)	4.58 (3.32)
Godin Leisure-time Activity Score			
% with score of 0	44	4	2
Quartiles	0, 9, 21	18, 33, 45	21,39, 63
Mean (SD)	13.98 (19.16)	33.54 (19.81)	43.68 (27.59)

Means and standard deviations for the SCT variables are presented in Table 3. A difference in exercise self-efficacy approached statistical significance (*p* = 0.05), and NHW had marginally higher self-efficacy (M = 26.0) compared to MA (M = 21.7; *p* = 0.076) and PR (M = 22.4, *p* = 0.164). Social support from family members differed among the groups (*p* = 0.052) and approached statistical significance, MA survivors reported more social support from family (M = 25.4) than did NHW (M = 22.4, *p* = 0.336) and PR survivors reported the least social support from family (M = 20.4; p = 0.055). Significant differences were apparent among the groups in their reporting of exposure to modeling of PA (p = 0.034) with PR survivors reporting significantly higher levels of modeling (M = 3.3) than did NHW (M = 2.22, p = 0.036) and higher levels of modeling than did MA (M = 2.64, *p* = 0.272). When we controlled for age, education, and employment status, the group differences for exercise self-efficacy and social support from family were somewhat attenuated (p = 0.286 and 0.118, respectively), whereas the effect of modeling remained statistically significant (p = 0.044).

**Table 3:** Comparisons of SCT variables assessed in Puerto Rican, Mexican-American and Non-Hispanic white breast cancer survivors

	Overall, M (SD)	PR, M (SD)	MA, M (SD)	NHW, M (SD)	ANOVA, *p*	Post-hoc, *‘t’*
Barriers self-efficacy	38.6 (12.5)	36.9 (13.9)	38.5 (9.1)	40.5 (13.8)	0.370	
Exercise self-efficacy	23.4 (9.4)	22.4 (11.2)	21.7 (7.6)	26.0 (8.7)	0.052	NHW > MA (*p* = 0.076) NHW > PR (*p* = 0.164)
Social support-friends	20.8 (12.0)	18.7 (10.8)	23.5 (13.9)	20.2 (10.6)	0.120	
Social support-family	22.7 (10.4)	20.4 (11.5)	25.4 (9.7)	22.3 (9.4)	0.052	MA > PR (*p* = 0.055) MA > NHW (*p* = 0.336)
Modeling	2.73 (2.14)	3.32 (2.37)	2.64 (2.07)	2.22 (1.87)	*0.034*	PR > NHW (*p* = 0.036) PR > MA (*p* = 0.272)
GLTEQ	28.4 (23.9)	12.9 (17.6)	31.4 (18.7)	40.9 (25.9)	*<0.001*	NHW > PR (p < 0.001) MA > PR (p < 0.001) NHW > MA (*p* = 0.083)

Italic values are statistically significant (*p* < 0.05).

*PR* Hispanic Puerto Rican participants, *MA* Hispanic Mexican-American participants, *NHW* non-Hispanic white participants, *M* mean, *SD* standard deviation, *GLTEQ* Godin Leisure Time Exercise Questionnaire Score.

In the regression model, predicting GLTEQ score with the predictor variables of ethnicity, age, and education (step 1), and exercise barriers self-efficacy, exercise self-efficacy, social support from family, social support from friends, and modeling entered as predictors (step 2), demographic variables predicted 26% of the variance in GLTEQ score (F = 9.9 [5,142], p < 0.001) and step 2 (SCT) variables predicted an additional 17% of the variance (F = 8.2 [5,137], p < 0.001) for a total explained variance of 43%. Significant predictors in this model were MA ethnicity (β = −0.18, t = −2.1, p = 0.040), PR ethnicity (β = −0.54, t = −6.5, p < 0.001), exercise barriers self-efficacy (β = 0.21, t = 2.6, p = 0.012), and modeling (β = 0.22, t = 2.8, p = 0.005).

When the interaction terms between ethnic group and each SCT variable were added to the model one SCT variable at a time, the only interactions that were significant were those between ethnic group and exercise barriers self-efficacy, and this explained an additional 4% of the variance in GLTEQ score (F-change = 4.6 [2,135], p = 0.012). In this model, the interactions between exercise barriers self-efficacy and MA (beta = −0.77, t = −2.7, p = 0.007) and PR (beta = −0.51, t = −2.3, p = 0.024) ethnicity were significant, indicating that exercise barriers self-efficacy was less predictive of GLTEQ score for MA and PR than it was for NHW.

## Discussion

Of the more than 12 million cancer survivors in the United States, approximately 15% are Hispanic (American Cancer Society 2009). The Latino population is the most rapidly growing ethnic group in the United States (United States Census Bureau 2000; Census Bureau 2011). As current trends continue, the number of Latina women with breast cancer in the US population will escalate just as dramatically (American Cancer Society 2009).

Consistent with published studies in Hispanic populations not affected by cancer (Cantero et al. 1999; Abraido-Lanza et al. 2005), our study indicates that Hispanic breast cancer survivors are less physically active than are non-Hispanic whites. Our study also was consistent with published studies that have shown higher BMI and waist-to-hip ratios for Latinas versus non-Hispanic whites (Hubert et al. 2005; Ogden et al. 2006; Slattery et al. 2006).

The percentage of breast cancer survivors reporting no moderate- or vigorous-intensity physical activity ranges from 18 to 76%, depending on ethnic group. The inactivity rates of PR survivors were markedly higher than those of the MA and NHW survivors. This is in contrast to data from the Behavioral Risk Factor Surveillance System indicating that rates of no exercise in the past 30 days are similar in Texan and Puerto Rican Hispanics (approximately 38%). In this survey, the Puerto Rican survivors’ median GLTEQ index was 9.0 (25th percentile = 0, 75th percentile = 21) (Center for Disease Control and Prevention 2012), indicating a very low level of current physical activity. The fact that 76% of our PR survivors indicate no moderate- to vigorous-intensity activity indicates that only a small proportion (<24%) of the respondents are meeting American Cancer Society physical activity guidelines for cancer survivors (Rock et al. 2012). This level of physical activity is lower than that reported by breast cancer survivors meeting physical activity recommendations in a survey of cancer survivors from a cross-sectional survey of cancer survivors in 16 state cancer registries (American Cancer Society’s Study of Cancer Survivors-II; ACS SCS-II). In this study (which had fewer than 10% Hispanic participants), 37% of the breast cancer survivors were meeting physical activity recommendations (Blanchard et al. 2008).

These very low activity levels could be attributable to several factors. In focus groups conducted with Puerto Rican and Mexican-American breast cancer survivors, we found that both groups lacked knowledge of the safety of physical activity after breast cancer. Although participants reported being physically active before their diagnosis, many reported that they did not know what they could or could not do after treatment. Focus group members expressed a desire for more information but few reported receiving any guidance or direction from health care providers with regard to physical activity. This situation was seemingly validated by modeling, as many of the breast cancer survivors reported being unaware of other cancer survivors engaging in exercise. In addition, many of our focus group participants reported that their family members did not encourage them to engage in exercise (Trevino et al. 2012). We also found that participants lacked knowledge about the benefits of physical activity; the Puerto Rican focus groups, in particular, mentioned several times that they did not think that physical activity could help prevent cancer. Because they did not see a link between physical activity and cancer, being active may have had less salience for the Puerto Rican survivors (Trevino et al. 2012).

Additionally, both groups reported a variety of other barriers to exercise. When asked about safety in their neighborhoods, Puerto Ricans stating they had little or no safety concerns, whereas the Mexican-American groups did report safety concerns as a barrier for physical activity. Both groups also mentioned social support as a barrier for physical activity, with Puerto Ricans stating that they would feel more motivated to be active if they had someone to exercise with that someone not necessarily having to be a family member (Trevino et al. 2012).

Furthermore, the PR survivors were recruited for this study in a public hospital in metropolitan San Juan Puerto Rico, and the MA and NHW participants were recruited through MD Anderson Cancer Center and from support groups in the Houston area. It is possible that the PR survivors were of lower socioeconomic status (SES), and this might help explain the lower rates of physical activity, although the differences in activity levels were not attenuated by controlling for education.

Our study also indicated that the activity-related SCT variables differed between Hispanic breast cancer survivor groups and non-Hispanic whites as has been reported in other studies (Whitehorse et al. 1999), but we also found a difference within Hispanic groups. MA survivors reported being less confident that they would be able to complete walking sessions of various lengths than did NHW survivors, and PR survivors reported seeing more people in their environment doing physical activity than did MA survivors. This difference may relate back to neighborhood safety issues in the MA group. Another difference between the two Hispanic groups was that social support for physical activity from family was higher in MA than in the PR women. This could be attributable to different cultural norms about exercise for cancer survivors in the two locations, although more research is needed to understand these cultural norms in promoting physical activity.

In associations between the SCT variables and leisure time physical activity, the set of five SCT variables predicted an additional 17% of the variance in the physical activity score. This percentage was above and beyond the effect of the demographic variables alone. Exercise barriers self-efficacy and modeling were significantly associated with activity levels. However, it should be noted that the association of barrier self-efficacy with activity was significantly smaller in the two Hispanic groups. Studies in other cancer survivor populations also have found associations between exercise barriers self-efficacy and physical activity, but these studies did not include large numbers of Hispanic survivors (Rogers et al. 2005; Bennett et al. 2007; Jones et al. 2005; Mosher et al. 2008; Pinto et al. 2009; Vallance et al. 2008).

Additional research is needed to determine why Hispanic survivors lack the confidence that one can overcome barriers to being consistently active and why they may believe exercise is less important. It is also important to understand how this information can be used further in culturally adapting interventions. The finding that modeling is a correlate of leisure time physical activity in these breast cancer survivors is somewhat novel, as this variable has not been routinely measured in many studies. A notable exception is a study by Rogers and colleagues, which showed that having an exercise role model was associated with steps taken and caloric expenditure in a sample of 21 breast cancer survivors receiving treatment (Rogers et al. 2005).

This study has certain limitations that should be noted. Because the sample size is modest, some of the lack of significant differences among the three groups in the SCT variables may be due to a lack of statistical power. The recruitment setting in Puerto Rico differed from that of the Mexican-American and non-Hispanic white populations and this may have accounted for some of the differences observed. We controlled for education level as a measure of SES status, but future studies should use additional measures of SES. In addition, because the sample is cross-sectional, the associations between the SCT variables and activity may not be causal. Further research is necessary to determine whether providing models for activity and improving barriers self-efficacy are effective tools for increasing physical activity in Hispanic breast cancer survivors. However, this study does represent one of the first attempts to measure and analyze social cognitive theory variables and leisure time physical activity in a sample of breast cancer survivors from two different Hispanic cultures. This study also had a comparison group of non-Hispanic white survivors that provided important information about future research directions.

## Conclusions

Further research aimed at understanding culturally specific variables of exercise behaviors for ethnic breast cancer survivors is warranted. Culturally adapted intervention strategies are needed for increasing physical activity in specific ethno-cultural populations.

## Abbreviations

PA: physical activity
PR: Puerto Rican
MA: Mexican-American
NHW: non-Hispanic white
SCT: social cognitive theory
GLTEQ: Godin Leisure Time Exercise Questionnaire
BMI: body mass index
W:H: waist-to-hip
M: mean
SD: standard deviation
BRTC: Behavioral Research and Treatment Center
UTMDACC: University of Texas M. D. Anderson Cancer Center
UPRCC: University of Puerto Rico Cancer Center
MET: metabolic equivalent
SPSS: statistical package for the social sciences
ANOVA: analysis of variance
SES: socioeconomic status
PROSPR: patient-reported outcomes, survey, and population research
